# The relationship between nutrition status indicators and vitamin D deficiency in patients with type 2 diabetes mellitus

**DOI:** 10.3389/fnut.2026.1777757

**Published:** 2026-04-24

**Authors:** Hang Zhao, Yunjia Zhang, Zhimei Zhang, Huanxin Liu, Luping Ren, Shuchun Chen

**Affiliations:** 1 Department of Endocrinology, Hebei General Hospital, Shijiazhuang, Hebei, China; 2 Health Examination Center, Hebei General Hospital, Shijiazhuang, Hebei, China

**Keywords:** nutrition, prealbumin, total protein, type 2 diabetes mellitus, vitamin D deficiency

## Abstract

**Purpose:**

To investigate the association between nutrition status indicators and vitamin D deficiency in type 2 diabetes mellitus (T2DM) patients.

**Patients and methods:**

This cross-sectional study enrolled 285 hospitalized T2DM patients from Hebei General Hospital. Vitamin D deficiency was defined as serum 25-hydroxyvitamin D (25OHD) < 20 ng/mL. Nutritional indicators, including total protein, albumin, prealbumin, body mass index (BMI), uric acid (UA), hemoglobin (Hb), and other information, were collected from medical records. Logistic regression, subgroup, and sensitivity analyses were used to explore the relationship between nutrition indicators and vitamin D deficiency.

**Results:**

The prevalence of vitamin D deficiency was 62% (178/285). Compared with the no vitamin D deficiency group, the vitamin D deficiency group had significantly lower total protein (67.4 ± 5.9 vs. 69.4 ± 6.1 g/L, *p* = 0.007), while the other indicators showed no difference between the two groups. After adjusting all confounding variables, a negative association existed between total protein and vitamin D deficiency (OR = 0.933, 95% CI: 0.891–0.975, *p* = 0.002). Subgroup analysis indicated that in patients aged <60 years (OR = 0.928, 95% CI: 0.873–0.987, *p* = 0.018), male patients (OR = 0.912, 95% CI: 0.863–0.965, *p* = 0.001), and those with diabetes for less than 10 years (OR = 0.935, 95% CI: 0.890–0.982, *p* = 0.007), elevated total protein was significantly associated with lower odds of vitamin D deficiency. For prealbumin, a similar association was observed in patients aged <60 years. Sensitivity analysis showed total protein (treated as tertiles) was negatively correlated with vitamin D deficiency (OR = 0.453, 95% CI: 0.281–0.721, *p* = 0.001). A second sensitivity analysis, using <12 ng/mL as the cutoff to define severe vitamin D deficiency, showed a consistent result (OR = 0.939, 95% CI: 0.887–0.991, *p* = 0.026).

**Conclusion:**

The nutrition indicator (total protein) was negatively related to vitamin D deficiency.

## 1 Introduction

Type 2 diabetes mellitus (T2DM) represents a global health crisis, affecting over 537 million adults worldwide, with its prevalence projected to escalate by 46% by 2045 (1). Generally speaking, unhealthy dietary habits and excessive calorie intake are risk factors for the development and progression of metabolic diseases such as obesity, T2DM, and non-alcoholic fatty liver disease. The first step in treatment is lifestyle interventions, including adjusting dietary patterns and controlling calorie intake. However, excessive calorie restriction and malnutrition can lead to severe consequences. For example, a prospective cohort study from the UK Biobank found serum albumin levels were inversely associated with the incidences of diabetes and diabetic microvascular complications. Among non-diabetic participants, with a median follow-up of 12.90 years, each 10 g/L increase in serum albumin was associated with a 12% lower incidence of diabetes. Among those with diabetes, per 10 g/L increase in serum albumin, the hazard ratios (HRs) for diabetic nephropathy, ophthalmopathy, and neuropathy were 0.42, 0.61, and 0.67, respectively (2). Another cohort study involving patients with T2DM in Japan found that poorer nutritional status (assessed by the Geriatric Nutritional Risk Index) was significantly associated with an increased risk of renal function decline (HR = 5.15, 95% confidence interval [CI]: 2.51–10.6) and all-cause mortality (HR = 2.30, 95% CI: 1.20–4.42) after a median follow-up of 4.5 years (3). Additionally, malnutrition not only increases the risk of osteoporosis (OP) (4) but also elevates mortality risk among individuals with existing OP (5).

Vitamin D deficiency is highly prevalent in clinical practice. It not only links to the onset of T2DM (6), but the proportion of individuals with T2DM who are deficient in vitamin D is also higher than that in other populations. It exacerbates metabolic dysregulation via impairing insulin sensitivity and *β*-cell function, while hyperglycemia and obesity reduce vitamin D bioavailability through adipose sequestration and altered hepatic metabolism (7–10). Critically, bidirectional interactions exist between nutritional status and vitamin D deficiency: obesity (11) diminishes vitamin D levels, whereas protein-energy malnutrition (reduced albumin/prealbumin) disrupts vitamin D transport via decreased synthesis of vitamin D-binding protein (12). In addition, it is closely associated with OP, cardiovascular disease, cancer, respiratory diseases, and mortality (13, 14).

Nutrition status can be assessed from various aspects, including anthropometric measurements, laboratory tests, and clinical signs. This study therefore aimed to comprehensively evaluate the relationship between simple nutritional indicators and vitamin D deficiency in T2DM, providing new insights of both nutritional and metabolic disorders in this population.

## 2 Patients and methods

### 2.1 Study design

We performed a cross-sectional study of eligible patients with T2DM who were hospitalized in the Endocrinology Department of Hebei General Hospital. The study adhered to the Declaration of Helsinki guidelines and was approved by the Ethics Committee of Hebei General Hospital (20250230). All participants signed informed consent. The Clinical Trial Registry Number is ChiCTR2000029391^1^ and registry data is 2020/01/29.

### 2.2 Inclusion and exclusion criteria

*Inclusion criteria*: (i) adults (≥18 years), (ii) fulfilment of the T2DM diagnostic criteria of the World Health Organization, 1999.

*Exclusion criteria*: (i) other types of diabetes; (ii) history of fractures; (iii) use of supplements of vitamin D or calcium; (iv) use of corticosteroids or immunosuppressants; (v) recent acute cardiovascular and cerebrovascular events, or recent surgery (≤3 months); (vi) chronic diarrhoea; (vii) rheumatic diseases.

### 2.3 Data collection

For eligible patients, the following data were collected.

(i) Basic information: Age, age group (<60 years, ≥60 years), gender (male, female), smoking history, drinking history, family history, hypertension history, diabetes course group (<10 years, ≥10 years).
(ii) Biochemical data: glycated hemoglobin (HbA1c), serum urea nitrogen (BUN), creatinine (Cr), total cholesterol (TC), triglycerides (TG), low-density lipoprotein-cholesterol (LDL-C), high-density lipoprotein-cholesterol (HDL-C), 25-hydroxyvitamin D (25OHD), osteocalcin (OC), procollagen type 1 N-terminal propeptide (P1NP), *β*-C-terminal cross-linked telopeptide of type I collagen (β-CTX), parathyroid hormone (PTH).
(iii) Nutrition indicators: total protein, albumin, prealbumin, body mass index (BMI), uric acid (UA), hemoglobin (Hb).

The patients were divided into two groups according to 25OHD. Vitamin D deficiency was defined as 25OHD < 20 ng/mL and no vitamin D deficiency was defined as ≥20 ng/mL, according to the Endocrine Society Clinical Practice Guideline (15) and the Chinese consensus on vitamin D deficiency (16).

### 2.4 Statistical analysis

SPSS 22.0 and R 4.5.1 software were used for statistical analysis. Continuous variables are expressed as the mean ± standard deviation for normally distributed data or median (P25, P75) for nonnormally distributed data. Categorical variables are presented as count (percentage). Independent samples *t* test (normally distributed continuous variables), nonparametric tests (nonnormally distributed continuous variables) and Chi-square test (categorical variables) were applied in comparisons between two groups. Binary logistic regression analysis was used to assess the association between nutrition indicators and vitamin D deficiency. Three models were fitted for the outcomes. In model 1 (crude model), no confounding variables were adjusted. In model 2, gender was adjusted for. Model 3 was further adjusted by including total protein, Cr, TG, and HbA1c. Subgroup analyses were conducted according to age (<60 years old and ≥60 years), gender (female and male), diabetes course (<10 years old and ≥10 years). Sensitivity analysis was used to assess the robustness of the results. First, nutrition indicators were categorized into tertiles [except for BMI groups (normal weight, overweight and obesity)] and repeated the regression analysis (in three models). Second, we performed an additional sensitivity analysis by redefining vitamin D deficiency as serum 25OHD < 12 ng/mL (severe deficiency) (17). Logistic regression analyses were then repeated using the same three models. *p* < 0.05 was regarded as indicating statistical significance.

## 3 Results

The study included 285 T2DM patients (187 male, 98 female) aged 23–84 years. Mean serum 25OHD was 17.8 (13.3, 23.2) ng/mL, with 178 (62%) classified as vitamin D deficiency (<20 ng/mL) and 107 (38%) as no vitamin D deficiency (≥20 ng/mL).

### 3.1 Comparisions between groups

Compared with no vitamin D deficiency group, vitamin D deficiency group had significantly fewer males (60.1% vs. 74.8%, *p* = 0.012), higher HbA1c [9.1 (7.8, 10.8) vs. 8.2 (6.8, 10.2) %, *p* = 0.003] and TG [1.5 (1.1, 2.4) vs. 1.4 (1.0, 1.7) mmol/L, *p* = 0.041]. Vitamin D deficiency group also showed lower Cr [69.6 (61.2, 78.5) vs. 74.6 (66.4, 83.0) μmol/L, *p* = 0.003], OC [11.7 (9.6, 14.7) vs. 13.3 (10.3, 18.1) ng/mL, *p* = 0.009], *β*-CTX [0.3 (0.2, 0.5) vs. 0.4 (0.2, 0.6) ng/mL, *p* = 0.036] and P1NP [36.1 (28.6, 46.5) vs. 40.2 (31.0, 53.8) ng/mL, *p* = 0.049]. Regarding nutrition indicators, vitamin D deficiency group showed lower total protein (67.4 ± 5.9 vs. 69.4 ± 6.1 g/L, *p* = 0.007), while the other indicators showed no difference between two groups (Table 1).

**Table 1:** Clinical characteristics of patients with T2DM by 25OHD category.

Characteristic	Overall (*n* = 285, 100%)	No deficiency (*n* = 107, 38%)	Deficiency (*n* = 178, 62%)	*p*-value
Age (years)	55.5 ± 11.9	55.8 ± 11.3	55.3 ± 12.4	0.721
Age group (*n*, %)				0.841
<60 years	175 (61.4%)	67 (62.6%)	108 (60.7%)	
≥60 years	110 (38.6%)	40 (37.4%)	70 (39.3%)	
Gender group (*n*, %)				0.017
Female	98 (34.4%)	27 (25.2%)	71 (39.9%)	
Male	187 (65.6%)	80 (74.8%)	107 (60.1%)	
Smoking history (*n*, %)	95 (33.7%)	41 (39.0%)	54 (30.5%)	0.181
Drinking history (*n*, %)	76 (26.9%)	29 (27.6%)	47 (26.4%)	0.933
Family history (*n*, %)	103 (36.4%)	34 (32.4%)	69 (38.8%)	0.342
Hypertension history (*n*, %)	125 (44.0%)	54 (50.9%)	71 (39.9%)	0.091
Course group (*n*, %)				>0.999
<10 years	247 (87.6%)	92 (87.6%)	155 (87.6%)	
≥10 years	35 (12.4%)	13 (12.4%)	22 (12.4%)	
HbA1c (%)	8.8 (7.4, 10.6)	8.2 (6.8, 10.2)	9.1 (7.8, 10.8)	0.003
TC (mmol/L)	4.7 ± 1.1	4.7 ± 1.0	4.7 ± 1.2	>0.999
TG (mmol/L)	1.5 (1.1, 2.2)	1.4 (1.0, 1.7)	1.5 (1.1, 2.4)	0.041
HDL-C (mmol/L)	1.0 ± 0.2	1.1 ± 0.2	1.0 ± 0.2	0.233
LDL-C (mmol/L)	3.1 ± 0.8	3.1 ± 0.7	3.1 ± 0.9	0.666
BUN (mmol/L)	5.3 ± 1.4	5.5 ± 1.2	5.2 ± 1.4	0.071
Cr (μmmol/L)	71.9 (62.8, 80.9)	74.6 (66.4, 83.0)	69.6 (61.2, 78.5)	0.003
25OHD (ng/mL)	17.8 (13.3, 23.2)	25.2 (22.0, 29.3)	14.4 (11.6, 17.1)	<0.001
OC (ng/mL)	12.1 (9.7, 16.0)	13.3 (10.3, 18.1)	11.7 (9.6, 14.7)	0.009
CTX (ng/mL)	0.3 (0.2, 0.5)	0.4 (0.2, 0.6)	0.3 (0.2, 0.5)	0.036
P1NP (ng/mL)	37.8 (29.4, 50.6)	40.2 (31.0, 53.8)	36.1 (28.6, 46.5)	0.049
PTH (pg/mL)	34.3 (25.0, 45.1)	32.3 (24.1, 43.6)	36.2 (26.2, 46.7)	0.109
Serum calcium (mmol/L)	2.3 (2.2, 2.4)	2.3 (2.2, 2.4)	2.3 (2.2, 2.4)	0.618
Nutrition status indicators				
Total protein (g/L)	68.1 ± 6.1	69.4 ± 6.1	67.4 ± 5.9	0.007
Albumin (g/L)	41.3 ± 3.2	41.7 ± 3.2	41.0 ± 3.2	0.106
Prealbumin (mg/L)	23.6 ± 5.4	24.2 ± 5.6	23.1 ± 5.3	0.080
BMI (kg/m^2^)	25.9 ± 3.4	25.4 ± 3.7	26.1 ± 3.2	0.116
UA (μmmol/L)	309.4 ± 82.5	311.7 ± 78.3	308.1 ± 85.1	0.862
Hb (g/L)	143.2 ± 16.5	143.6 ± 16.3	143.0 ± 16.6	0.607

Data were expressed as mean ± SD/median (P25, P75) or number (%).

BMI, body mass index; BUN, blood urea nitrogen; Cr, creatinine; HbA1c, glycated hemoglobin; Hb, hemoglobin; HDL-C, high-density lipoprotein cholesterol; LDL-C, low-density lipoprotein cholesterol; OC, osteocalcin; P1NP, procollagen type 1 N-terminal propeptide; PTH, parathyroid hormone; T2DM, type 2 diabetes mellitus; TC, total cholesterol; TG, triglyceride; UA, uric acid; 25OHD, 25-hydroxyvitamin D; β-CTX, β-C-terminal cross-linked telopeptide of type I collagen.

### 3.2 Regression analysis

In Model 1 (crude model), the increase of total protein is significantly related to lower odds of vitamin D deficiency (OR = 0.947, 95% CI: 0.908–0.985, *p* = 0.008). After adjusting for gender (Model 2), the association enhanced (OR = 0.940, 95% CI: 0.901–0.980, *p* = 0.004). After adjusting HbA1c, TG, Cr and OC based on Model 2, there remained a negative association between total protein and vitamin D deficiency (OR = 0.933, 95% CI:0.891–0.975, *p* = 0.002).

In Model 1, Cr was negatively associated with vitamin D deficiency (OR = 0.977, 95% CI 0.960–0.994, *p* = 0.010), but after adjusting confounding variables, there was no association (OR = 0.990, 95% CI: 0.968–1.012, *p* = 0.350).

Albumin, prealbumin, BMI, UA, and Hb did not demonstrate significant associations with vitamin D deficiency in the three models (All *p* > 0.05) (Table 2).

**Table 2:** Association of nutrition indicators (as continuous variable) and vitamin D deficiency.

Nutrition status indicators	Model 1		Model 2		Model 3	
	OR (95% CI)	*p*-value	OR (95% CI)	*p*-value	OR (95% CI)	*p*-value
Total protein	0.947 (0.908, 0.985)	0.008	0.940 (0.901, 0.980)	0.004	0.933 (0.891, 0.975)	0.002
Albumin	0.935 (0.866, 1.008)	0.083	0.950 (0.878, 1.027)	0.198	1.067 (0.952, 1.198)	0.267
Prealbumin	0.963 (0.920, 1.008)	0.105	0.965 (0.921, 1.011)	0.133	0.960 (0.960, 1.129)	0.339
BMI	1.105 (0.988, 1.142)	0.077	1.069 (0.994, 1.152)	0.071	1.042 (0.962, 1.130)	0.321
UA	0.999 (0.997, 1.002)	0.717	1.000 (0.997, 1.004)	0.780	1.002 (0.998, 1.005)	0.381
Hb	0.998 (0.983, 1.012)	0.742	1.010 (0.993, 1.028)	0.245	1.010 (0.990, 1.030)	0.331

The results were expressed as OR and 95% CI. Model 1, no confounding factors were adjusted. Model 2, gender was adjusted. Model 3, gender, HbA1c, TG, Cr, OC and total protein were adjusted.

BMI, body mass index; Cr, creatinine; HbA1c, glycated hemoglobin; Hb, hemoglobin; OC, osteocalcin; TG, triglyceride; UA, uric acid.

### 3.3 Subgroup analysis

**Figure 1:**
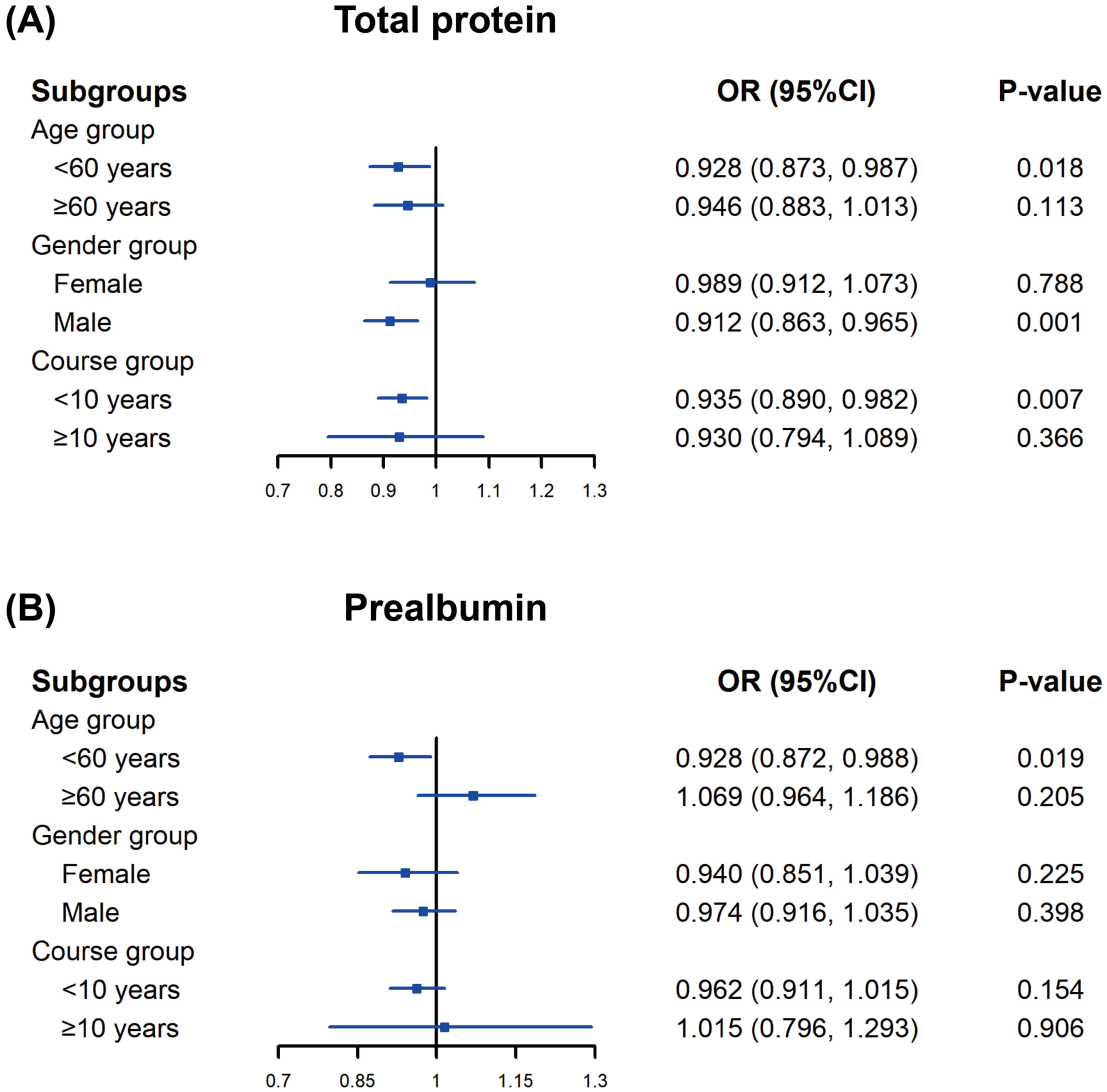
Subgroup analysis of the association between nutrition status indicators and vitamin D deficiency. **(A)** Total protein, **(B)** Prealbumin.

We conducted subgroup analyses based on age, gender and diabetes course. In patients aged <60 years (OR = 0.928, 95% CI: 0.873–0.987, *p* = 0.018), male patients (OR = 0.912, 95% CI: 0.863–0.965, *p* = 0.001), and those with diabetes course <10 years (OR = 0.935, 95% CI: 0.890–0.982, *p* = 0.007), elevated total protein was significantly associated with reduced odds of vitamin D deficiency. For prealbumin, a similar association was observed in patients aged <60 years (Figure 1).

No significant associations were found for other indicators (Albumin, BMI, UA and Hb) across all subgroups (Supplementary Figure S1).

### 3.4 Sensitivity analysis

To verify the robustness of the results, we categorized the nutrition indicators into tertiles and conducted regression analyses. The results were consistent with our previous findings: total protein was negatively correlated with vitamin D deficiency (OR = 0.453, 95% CI: 0.281–0.721, *p* = 0.001) and no such correlation was observed for other indicators (Table 3).

**Table 3:** Association of nutrition indicators (as tertiles variable) and vitamin D deficiency.

Nutrition status indicators	Model 1		Model 2		Model 3	
	OR (95% CI)	*p*-value	OR (95% CI)	*p*-value	OR (95% CI)	*p*-value
Total protein	0.511 (0.329, 0.783)	0.002	0.484 (0.309, 0.747)	0.001	0.453 (0.281, 0.721)	0.001
Albumin	0.738 (0.482, 1.123)	0.158	0.805 (0.522, 1.237)	0.323	1.472 (0.811, 2.712)	0.208
Prealbumin	0.963 (0.920, 1.008)	0.105	0.965 (0.921, 1.011)	0.133	0.961 (0.914, 1.011)	0.126
BMI	1.601 (1.007, 2.578)	0.049	1.649 (1.031, 2.674)	0.039	1.410 (0.840, 2.387)	0.195
UA	1.033 (0.679, 1.571)	0.880	1.194 (0.772, 1.855)	0.426	1.502 (0.890, 2.568)	0.132
Hb	1.007 (0.662, 1.535)	0.972	1.606 (0.966, 2.712)	0.071	1.537 (0.884, 2.710)	0.131

The results were expressed as OR and 95% CI. Model 1, no confounding factors were adjusted. Model 2, gender was adjusted. Model 3, gender, HbA1c, TG, Cr, OC and total protein were adjusted.

BMI, body mass index; Cr, creatininel; HbA1c, glycated hemoglobin; Hb, hemoglobin; OC, osteocalcin; TG, triglyceride; UA, uric acid.

We further performed a sensitivity analysis using an alternative clinical threshold of <12 ng/mL to define severe vitamin D deficiency. Among the 285 patients, 50 (18%) had 25OHD < 12 ng/mL. The negative association between total protein and severe vitamin D deficiency remained statistically significant (OR = 0.939, 95% CI: 0.887–0.991, *p* = 0.026) after adjusting for confounding variables (Table 4).

**Table 4:** Association of nutrition indicators (as continuous variable) and vitamin D severe deficiency (25OHD < 12 ng/mL).

Nutrition status indicators	Model 1		Model 2		Model 3	
	OR (95% CI)	*p*-value	OR (95% CI)	*p*-value	OR (95% CI)	*p*-value
Total protein	0.943 (0.893, 0.993)	0.030	0.936 (0.885, 0.987)	0.016	0.939 (0.887, 0.991)	0.026
Albumin	0.868 (0.784, 0.956)	0.005	0.884 (0.798, 0.977)	0.016	0.968 (0.840, 1.120)	0.660
Prealbumin	1.013 (0.955, 1.071)	0.665	1.017 (0.959, 1.077)	0.563	1.014 (0.953, 1.077)	0.646
BMI	1.068 (0.977, 1.167)	0.147	1.073 (0.982, 1.173)	0.118	1.093 (0.989, 1.208)	0.080
UA	0.998 (0.994, 1.002)	0.418	1.000 (0.996, 1.004)	0.873	0.999 (0.995, 1.004)	0.781
Hb	0.986 (0.968, 1.005)	0.147	0.999 (0.977, 1.022)	0.910	0.997 (0.974, 1.022)	0.825

The results were expressed as OR and 95% CI. Model 1, no confounding factors were adjusted. Model 2, gender was adjusted. Model 3, gender, HbA1c, TG, Cr, OC and total protein were adjusted.

25OHD, 25-hydroxyvitamin D; BMI, body mass index; Cr, creatinine; HbA1c, glycated hemoglobin; Hb, hemoglobin; OC, osteocalcin; TG, triglyceride; UA, uric acid.

## 4 Discussion

This study revealed associations between nutritional status indicators and vitamin D deficiency in patients with T2DM. Total protein was negatively related to vitamin D deficiency. Unlike albumin, which can be suppressed by systemic inflammation (18), or prealbumin, which has a short half-life and reflects short-term nutritional changes (19), total protein provides a more integrative measure of protein nutritional status, including immunoglobulins and transport proteins. Given that vitamin D-binding protein (DBP) is synthesized in the liver, reduced total protein may directly impair DBP availability, thereby contributing to functional vitamin D deficiency (20, 21). This mechanistic link may explain why total protein, rather than albumin or prealbumin alone, showed a more consistent negative association with vitamin D deficiency in our study.

This cross-sectional study could not establish causality between total protein and vitamin D deficiency. Reverse causality by which vitamin D affects protein levels is also plausible. First, vitamin D regulates the expression of multiple genes involved in protein metabolism by activating the vitamin D receptor (VDR) in skeletal muscle nuclei. VDR activation suppresses the ubiquitin-proteasome pathway and the autophagy-lysosome pathway, thereby reducing degradation of myofibrillar proteins. Vitamin D deficiency attenuates this inhibition, leading to accelerated muscle protein breakdown (22, 23). Second, VDR signaling inhibits the release of inflammatory factors such as TNF-*α* and IL-6 and reduces the promoting effect of chronic inflammation on muscle protein breakdown (24, 25). Third, vitamin D maintains intestinal barrier integrity by upregulating the expression of tight junction proteins (e.g., claudin-1) in intestinal epithelial cells. Vitamin D deficiency impairs tight junctions and increases intestinal permeability, allowing pro-inflammatory factors to enter the circulation and exacerbating systemic inflammation, while also directly impairing protein absorption (26). Fourth, vitamin D enhances growth hormone (GH) receptor expression as well as the synthesis and secretion of insulin-like growth factor-1 (IGF-1), a key factor for protein synthesis (27). Therefore, the negative association may partly reflect the effect of vitamin D deficiency on protein levels. Future prospective cohort studies are needed to clarify this causality.

Subgroup analyses indicated difference. In patients aged <60 years, total protein and prealbumin had a negative relation with vitamin D deficiency, respectively. We speculate that this may be because sarcopenia and impaired digestive and/or absorptive functions are common in elderly patients with T2DM (28). Even when total protein levels appear normal, their intramuscular protein reserves may already be insufficient. At the same time, the capacity for vitamin D activation declines in older adults due to reduced kidney function. One hypothesis is that the association between total protein and vitamin D in this population may be obscured by other related factors, such as muscle loss and decreased renal function. In this study, the negative correlation between total protein and vitamin D deficiency existed in male patients, but not in females. A possible explanation is that male patients with T2DM have greater muscle mass (higher proportion of lean body mass), which might lead to higher protein intake requirements and more active protein metabolism. In addition, it is plausible that males have a lower risk of vitamin D deficiency compared with females, which may be related to the regulatory effect of estrogen on vitamin D (29). For T2DM patients with a shorter duration, one possible hypothesis is that the incidence of related complications is relatively low, and renal 1α-hydroxylase activity as well as intestinal absorption function are comparatively preserved. In contrast, patients with a longer duration often have reduced glomerular filtration rate, which may directly impair vitamin D activation. Patients with shorter disease duration are better able to reflect the direct link between “nutrition-vitamin D” in the early stage of the disease.

In the early stage of vitamin D deficiency, PTH elevation stimulates bone resorption. Progressive deficiency, however, suppresses bone formation. Coupled with the metabolic disorders related to diabetes (hyperglycemia, insulin resistance), both bone resorption and bone formation are eventually suppressed, resulting in a “low bone turnover state” (30). This mechanism explains why bone metabolism markers (OC, P1NP, *β*-CTX) in vitamin D deficient patients decrease simultaneously and the risk of fractures increases (31).

This study has some strengths. First, we included several simple indicators commonly used in clinical practice for roughly assessing nutrition status, rather than relying on a single indicator. Second, we conducted statistical analyses across different subgroups and the results were more clinically instructive. Third, the sensitivity analyses were conducted to verify the robustness of the results. We treated continuous variables as tertiles for statistical analysis, making the results more convincing. This study still has several limitations. First, due to the small sample size of this study, we did not conduct a dose–response analysis, so this study cannot determine whether there was a non-linear relationship between nutrition status indicators and vitamin D deficiency. Second, our study included hospitalized patients with T2DM. Compared with outpatients or community-dwellers, hospitalized patients have higher glucose levels and poorer nutrition, limiting our findings’ generalizability. We observed a negative association between total protein and vitamin D deficiency. Given that the biological mechanism linking total protein to vitamin D-binding protein synthesis is universal, we hypothesize that the direction of this association is likely to be consistent in non-hospitalized patients, although the strength of the association may be weaker due to their relatively better metabolic and nutritional status. Definitive conclusions require external validation in this population. Third, several potential unmeasured confounders were not collected or available in the study. Regarding antidiabetic medications, such as thiazolidinediones, biguanides, and SGLT-2 inhibitors, all have effects on bone metabolism. For example, SGLT-2 inhibitors may promote phosphate excretion, secondarily elevate PTH levels, and increase bone resorption. These agents have also been associated with altered vitamin D metabolism and reduced intestinal calcium absorption, influencing both bone turnover and vitamin D status (32, 33). In addition to medications, other variables include: detailed dietary habits (e.g., macronutrient composition, meal preferences); lifestyle factors (e.g., sun exposure and physical activity) and regular supplementation of vitamin D and calcium (including dose, frequency, and formulation). These factors may independently or interactively affect total protein levels, 25OHD levels, and their association. Because these variables are absent, residual confounding cannot be ruled out. Future prospective studies should collect them via detailed questionnaires to obtain more robust findings. Fourth, the limitation of the study design. This study was a single-center cross-sectional study, and the samples are only from one hospital in Hebei Province. There may be regional selection bias, and the generalizability of the research results was limited. The cross-sectional design cannot determine the causal relationship between nutrition indicators and vitamin D deficiency, but only reveals the correlation. Future studies need to verify the temporal relationship through longitudinal cohort studies.

## 5 Conclusion

In conclusion, total protein levels were negatively related to vitamin D deficiency. Subgroup analysis indicated that in patients aged <60 years, male patients and those with diabetes course <10 years, elevated total protein was significantly associated with reduced odds of vitamin D deficiency. For prealbumin, a similar association was observed in patients aged <60 years. It reminds us that for patients with T2DM, it is important to seek a balance between diet and blood glucose control, avoiding too strict eating that could lead to malnutrition and consequently result in vitamin D deficiency.

## Data Availability

The raw data supporting the conclusions of this article will be made available by the authors, without undue reservation.

## Footnotes

1
https://www.chictr.org.cn/showproj.html?proj=47050
