# Supplementary figures and images for: The relationship between nutrition status indicators and vitamin D deficiency in patients with type 2 diabetes mellitus
Source: Front Nutr. 2026 Apr 24;13:1777757. doi: 10.3389/fnut.2026.1777757 (PMC13152821; doi:10.3389/fnut.2026.1777757)

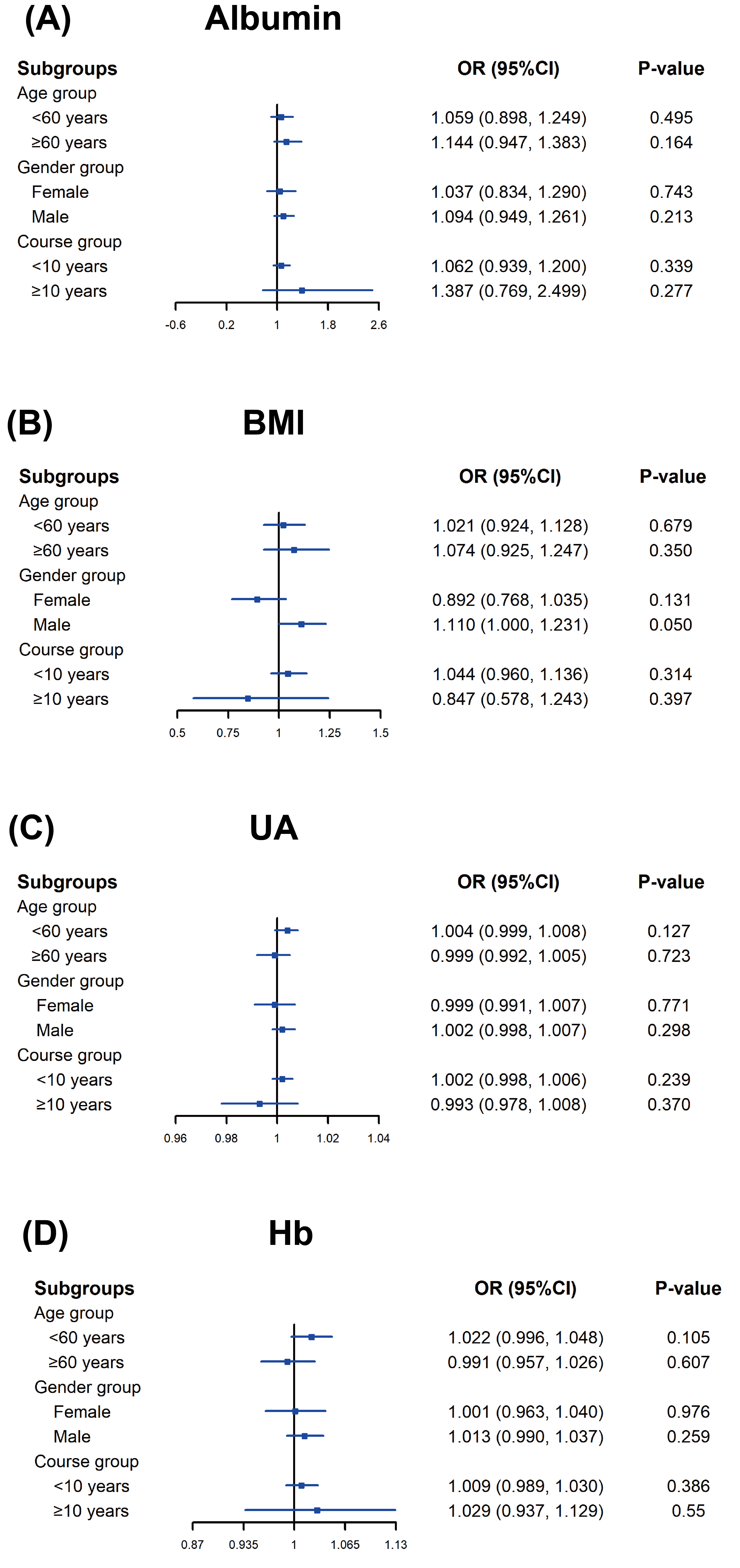

Supplement: Supplementary file 1 [file Image_1.jpeg]
